# Mo-Doped Cu_2_S Multilayer Nanosheets Grown In Situ on Copper Foam for Efficient Hydrogen Evolution Reaction

**DOI:** 10.3390/molecules27185961

**Published:** 2022-09-13

**Authors:** Yajie Xie, Jianfeng Huang, Rui Xu, Danyang He, Mengfan Niu, Xiaoyi Li, Guoting Xu, Liyun Cao, Liangliang Feng

**Affiliations:** 1 Key Laboratory of Auxiliary Chemistry and Technology for Chemical Industry, Ministry of Education, Shaanxi University of Science and Technology, Xi’an 710021, China; 2 School of Material Science and Engineering, International S&T Cooperation Foundation of Shaanxi Province, Shaanxi University of Science and Technology, Xi’an 710021, China

**Keywords:** Mo doping, Cu_2_S, electrocatalyst, hydrogen evolution

## Abstract

Metal sulfide electrocatalyst is developed as a cost-effective and promising candidate for hydrogen evolution reaction (HER). In this work, we report a novel Mo-doped Cu_2_S self-supported electrocatalyst grown in situ on three-dimensional copper foam via a facile sulfurization treatment method. Interestingly, Mo-Cu_2_S nanosheet structure increases the electrochemically active area, and the large fleecy multilayer flower structure assembled by small nanosheet facilitates the flow of electrolyte in and out. More broadly, the introduction of Mo can adjust the electronic structure, significantly increase the volmer step rate, and accelerate the reaction kinetics. As compared to the pure Cu_2_S self-supported electrocatalyst, the Mo-Cu_2_S/CF show much better alkaline HER performance with lower overpotential (18 mV at 10 mA cm^−2^, 322 mV at 100 mA cm^−2^) and long-term durability. Our work constructs a novel copper based in-situ metal sulfide electrocatalysts and provides a new idea to adjust the morphology and electronic structure by doping for promoting HER performance.

## 1. Introduction

Nowadays, in order to alleviate the non-renewable fossil energy and the associated serious environmental pollution, clean and sustainable energy has been vigorously pursued all over the world. Hydrogen gas, as a green, renewable, high calorific value of combustions and pollution-free energy carrier was widely concerned [1,2,3,4]. Among the current three main methods of hydrogen production, water electrolysis is considered to be an ideal pathway with zero-emission, high purity, and abundant water reserves. It is well known that noble metal platinum (Pt) and platinum-based materials offer the most efficient and stable electrocatalytic activity [5,6]. However, the scarcity and high cost of platinum (Pt) seriously limit its practical deployment. Thus, it is urgent to develop new high-performance and cost-effective electrocatalysts for hydrogen evolution reaction (HER).

Benefitting from the d-orbital electrons and vacant d-orbitals simultaneously existing in transition metal, it is easy to lose or capture electrons and has strong capable of redox reaction, which can bring down the activation energy of the water-splitting-related intermediates generated and thus promote the electrocatalytic process. Until now, transition metal-based electrocatalysts, including phosphides [7,8,9,10,11], (hydro) oxides [12,13], selenides [14,15], etc., have attracted much attention. Among them, transition metal sulfides (TMS) have been exploited by untiring effort as a considerably promising electrocatalyst in view of their advantages of economy and excellent electrochemical performance on water splitting [16,17,18,19]. Compared with Fe, Co and Ni, Cu was recognized as having favorable stability, excellent electrical conductivity and cost effectiveness; therefore, copper sulfide has been widely investigated in energy storage and conversion fields, such as battery [20,21], capacitor [22,23] and photocatalysis [24,25]. For example, Zhu et al. synthesized hierarchical cuprous sulfide nanosheets to modify nanowires on copper foam as a non-binder conversion cathode material for lithium/magnesium hybrid battery [20]. Li et al. successfully exploited multistage hybrid cuprous sulfide (Cu_2_S) nanoparticles anchoring on graphene to improve capacitive energy storage [22]. Wang et al. constructed cuprous sulfide nanoparticles for photocatalytic reduction of carbon dioxide on amorphous CuS_X_ matrix and successfully boosted the catalysis of cuprous sulfide with hybrid structure [24]. However, cuprous sulfide (Cu_2_S) is rarely used in the realm of electrochemical hydrogen evolution [26,27,28]. Zhang et al. proposed a “d-orbital complementarity” principle for the synthesis of vanadium-doped cobalt phosphide (V-CoP) as an efficient electrocatalyst towards HER [29]. Inspired by the “d-orbital complementarity” principle, we aim to develop a simple and universal method such as doping the early transition metal Mo to cuprous sulfide, which possesses the late transition metal copper. We aim to modulate the electronic structure and nano-structure, increase the specific surface area of catalysts, and enhance catalytic activity. Thus, the high-cost challenge may be alleviated, making it promising in use in large-scale and highly efficient water electrolysis.

In this report, molybdenum doped Cu_2_S multilayer nanosheets grown in situ on copper foam (CF) were synthesized by one-step hydrothermal route, denoted as Mo-Cu_2_S/CF. The results show that the molybdenum doping changed the nano-structure of the catalyst and formed a fluffy multilayer flower-like structure, leading to enlarging more active surface area, and promoted the optimization of electronic structure for improved intrinsic activity. The optimized Mo-Cu_2_S/CF exhibited extraordinarily low HER overpotential and robust stability in alkaline solution which only required 18 mV and 322 mV overpotential for obtaining a high current density of 10 and 100 mA cm^−2^, and worked stably for at least 20 h. Our work shows an innovative way to design and fabricate potential metal sulfide electrocatalytic materials for hydrogen production in water splitting.

## 2. Results and Discussion

**Figure 1:**
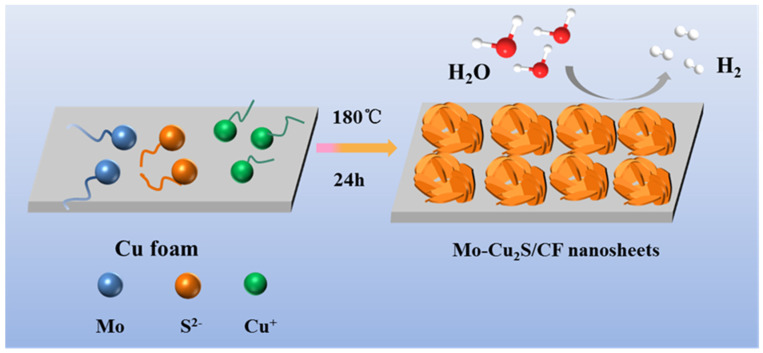
Schematic illustration of the construction of Mo-Cu_2_S/CF.

**Figure 2:**
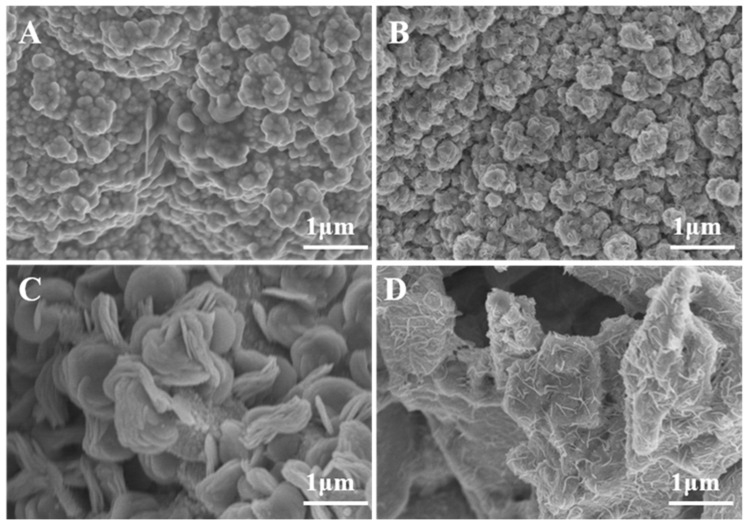
SEM images of Mo-Cu_2_S/CF (**A**) 6 h; (**B**) 12 h; (**C**) 24 h; (**D**) 36 h.

Molybdenum doped Cu_2_S multilayer nanosheets grown in situ on copper foam (Mo-Cu_2_S/CF) is fabricated by a facile one-step sulfurization hydrothermal method with sulfur source thioacetamide in a hydrothermal system at 180 °C for 24 h, as illustrated in Figure 1. The surface of CF was coarsened by sulfurization etching, which was more conducive to the growth of self-supported electrode, and the Cu^2+^ ions from CF are precipitated into the solution reacting with S^2-^ ions, meanwhile with the successful doping of molybdenum source, Mo-Cu_2_S nanosheet array was successfully grown on the CF. The SEM images in Figure 2 clearly reveal that hydrothermal reaction time has a significant impact on the structure and morphology of Mo-Cu_2_S/CF. When the hydrothermal reaction time is 6 h (Figure 2A), the CF surface is completely covered with the agglomerated balls. With the increase of reaction time 12 h (Figure 2B), small numbers of nanosheets can be observed on the surface of the agglomeration. Extending reaction time to 24 h (Figure 2C), the nanosheet with curved edge can be observed clearly. Obviously, the puffy multilayer nanosheet structure expands the contact area, facilitates the electron transfer rate and the electrolyte in and out. As the reaction time is prolonged to 36 h (Figure 2D), the nanosheets gradually disappear, which is not conducive to the electrolysis reaction to some extent. The phase of Mo-Cu_2_S/CF-6 h, Mo-Cu_2_S/CF-12 h, Mo-Cu_2_S/CF-24 h, Mo-Cu_2_S/CF-36 h remains Mo-Cu_2_S, as demonstrated in Figure S1.

**Figure 3:**
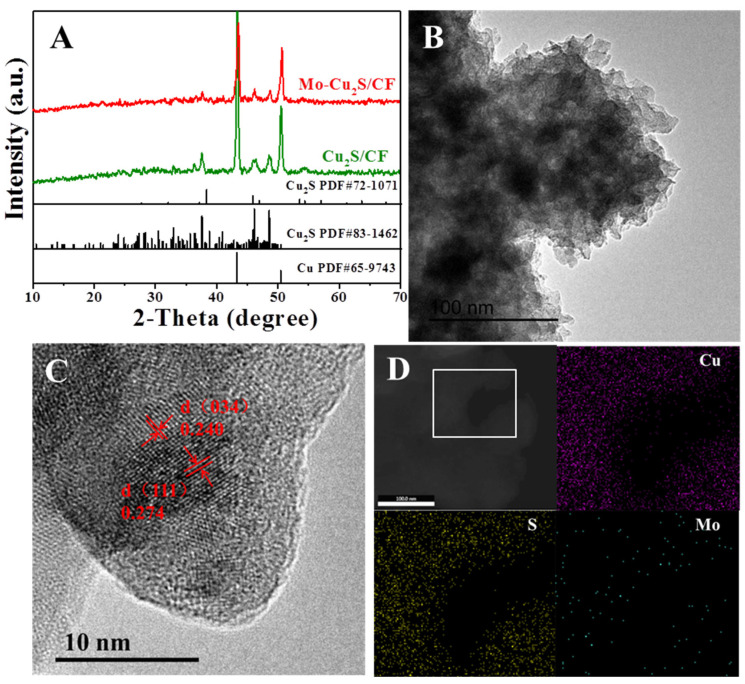
(**A**) XRD patterns of Mo-Cu_2_S/CF and Cu_2_S/CF; (**B**) TEM image; (**C**) the HRTEM image of Mo-Cu_2_S/CF; (**D**) the elemental mapping images.

Figure 3A displays the X-ray diffraction (XRD) spectra of Mo-Cu_2_S/CF and Cu_2_S/CF exhibited three typical diffraction peaks at 38.33°, 45.17°, 47.76° matches well with Cu_2_S (PDF#83-1462), and the two obvious peaks at 43.41° and 50.56° belong to the substrate Cu (PDF#65-9743). This reveals that Cu_2_S has good crystallinity, and an “all in one” structured Cu_2_S/CF is successfully synthesized. The TEM image of Mo-Cu_2_S/CF is observed in Figure 3B, Mo-Cu_2_S/CF is composed of many stacked nanosheet structures, which is consistent with SEM images. The HRTEM was also conducted in Figure 3C, a clear lattice spacing ~0.24 nm and ~0.274 nm corresponding to (111) and (034) planes of Cu_2_S (PDF#72-1071) and (PDF#83-1462) respectively consists well with the XRD results. Further, the EDS mapping images of Mo-Cu_2_S/CF clearly confirms that Cu, Mo, and S elements are uniformly dispersed over the entire area of nanosheet, resulting in the formation of Mo-doped Cu_2_S material (Figure 3D).

As a comparison, the SEM, TEM and HRTEM images of Cu_2_S/CF are performed in Supporting Information. The SEM image of Cu_2_S/CF (Figure S2A) displays a block structure with much larger size compared to Mo-Cu_2_S/CF, and this is evident in the TEM image (Figure S2B). To further determine the microstructure of Cu_2_S/CF, the HRTEM image of Cu_2_S/CF is conducted (Figure S2C), and visible lattice fringes with spacing of 0.274 nm are matched with Cu_2_S (111) plane. The corresponding elemental mapping images (EDX) of Cu_2_S/CF indicate that Cu and S are uniformly distributed over the whole Cu_2_S/CF, implying a homogeneous sulfurization (Figure S2D–F). By comparing Figure 3, Figures S2, S3 and Table S1, it is easy to draw a conclusion that Mo element was successfully induced and played a crucial role in the formation of nanosheet structure, reduced the size of Cu_2_S which is conducive to fully exposing active sites on the catalyst surface and making the hydrogen evolution process more efficient. Compared with massive block structures of Cu_2_S/CF, Mo-Cu_2_S/CF with the large fleecy multilayer flower-like morphologies assembled by asmall nanosheet obviously exposes more active sites and facilitates the flow of electrolyte in and out, effectively improving the efficiency of electrocatalytic hydrogen evolution [30].

**Figure 4:**
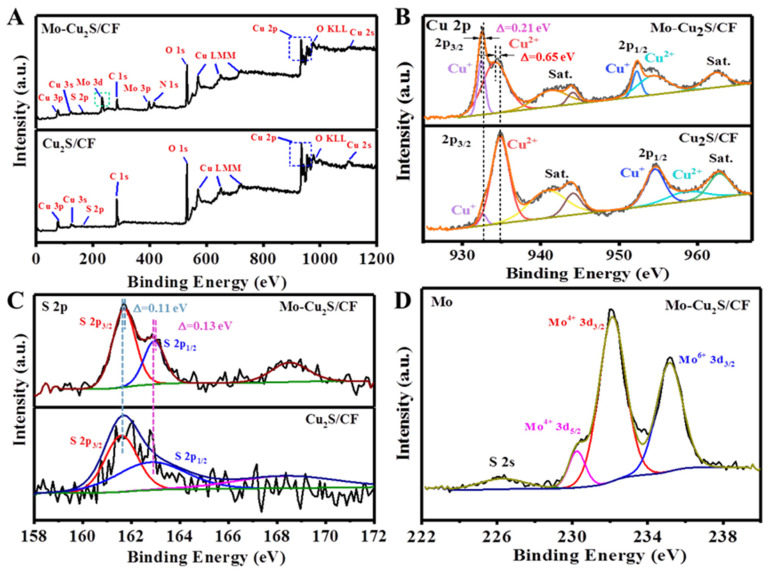
(**A**) Survey; (**B**) Cu 2p, (**C**) S 2p XPS spectra of Mo-Cu_2_S/CF and Cu_2_S/CF; (**D**) Mo XPS spectra of Mo-Cu_2_S/CF.

In addition, X-ray photoelectron spectroscopy (XPS) measurements were utilized to elucidate the electronic structure and chemical states on the surface of Mo-Cu_2_S/CF and Cu_2_S/CF samples. Cu, S, C, O elements of Mo-Cu_2_S/CF and Cu_2_S/CF are clearly observed in Figure 4A, while the Mo 3d signal appears in Mo-Cu_2_S/CF, indicating that Mo is successfully doped in the Cu_2_S/CF sample. Oxygen signal originates from the surface oxidation in the air. For the case of high-resolution of Cu 2p (Figure 4B), the spectrum clearly displays the Cu 2p_3/2_ peaks at 932.43 eV and 934.18 eV [31], which corresponds to Cu^+^ and Cu^2+^ states, respectively. The peaks of Cu 2p_1/2_ located at 952.28 and 954.34 eV belong to states of Cu^+^ and Cu^2+^. The satellite peaks at 941.32, 944.09 and 962.38 eV confirmed the existence of Cu^2+^, which is caused by exposure to air [32,33,34]. To be noticed, Cu 2p_3/2_ at 932.43 and 934.18 eV exhibit negative shifts of ~0.21, ~0.65 eV, respectively, compared with Cu_2_S/CF (952.28 and 954.34 eV), which could lead to the buildup of negative charges on and therefore favoring the adsorption of H* intermediates [35,36,37]. The in-situ Cu^2+^ reduction into Cu^+^ ions could be caused by S^2-^ ions reducing agents of Mo-Cu_2_S/CF [38]. Meanwhile, we observed that the characteristic peak of Cu^+^ 2p_3/2_ increases conspicuously in intensity along with the intensity area enlarging from 1.74% to 11.78% compared with Cu_2_S/CF. These results demonstrate that Mo-Cu_2_S/CF has better HER performance because Cu^+^ species have easier electron transfer than Cu^2+^ [27]. Figure 4C shows the comparison of S 2p XPS spectrum among Mo-Cu_2_S/CF and Cu_2_S/CF. There are two characteristic peaks with energy of 161.7 and 162.94 eV that can be assigned to S 2p_3/2_ and S 2p_1/2_ [39], indicating the formation of metal sulfides. The weak peak at 168.52 eV corresponds to SO_4_^2-^, which roots in the surface oxidation of Cu_2_S in the air [40]. The binding energies of S 2p peaks in Mo-Cu_2_S/CF have positive shifts of ~0.11, ~0.13 eV, as compared to Cu_2_S/CF (161.59 and 162.81 eV). Figure 4D demonstrates the successful synthesis of Mo-Cu_2_S/CF because the two characteristic peaks at 230.23 and 232.11 eV belong to Mo^4+^ 3d_5/2_ and Mo^4+^ 3d_3/2_, respectively. Additionally, the higher energy peak at 235.15 eV is indexed to Mo^6+^, because of the slight oxidation of the sample when exposed to air. From the binding energy deviation of Cu and S, we can conclude that electrons are transferred from S to Cu, indicating that the doping of Mo changes the electronic structure of Cu_2_S/CF successfully, and confirming strong electronic interaction between Mo and Cu_2_S, which can lead to enhancing electrical conductivity of the material [28,40]. The doping of molybdenum changes the content of Cu^+^, the increasing Cu^+^ is more conducive to hydrogen evolution reaction.

**Figure 5:**
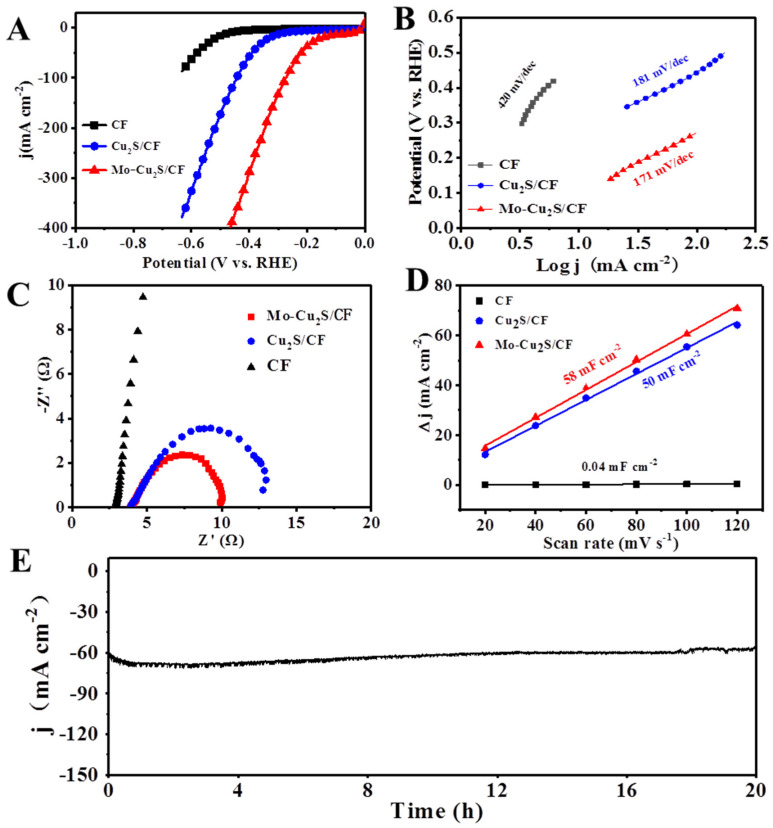
(**A**) LSV polarization curves of Mo-Cu_2_S/CF, Cu_2_S/CF and CF in1.0 M KOH at scan rate of 5 mVs^−1^; (**B**) Tafel curves; (**C**) EIS Nyquist plots; (**D**) C_dl_ plots for the estimation of the ECSA; (**E**) chronoamperometric curve (I-t) obtained for HER with Mo-Cu_2_S/CF at the current density of 60 mA cm^−2^ in 1 M KOH.

The electrocatalytic HER activity of Mo-Cu_2_S/CF was tested in Figure 5. Linear sweep voltammetry (LSV) measurements were recorded in 0.1M KOH solution at scan rate of 5 mV s^−1^. For comparison, the electrochemical hydrogen evolution tests of four samples with different hydrothermal times are carried out respectively (Figure S4). Mo-Cu_2_S/CF-24 exhibits best electrocatalytic activity towards HER, with quite a small overpotential of 18 and 322 mV to deliver a current density of 10 and 100 mA cm^−2^. In order to verify that the HER activity is driven by the catalytic site on Mo-Cu_2_S/CF, the blank CF and Cu_2_S/CF are tested respectively. As shown in Figure 5A, Mo-Cu_2_S/CF exhibits remarkable HER activity in alkaline media, which requires only a quite small overpotentials of 18/322 mV to deliver the current densities of 10/100 mA cm^−2^, and significantly outperforms Cu_2_S/CF (277/445 mV). It was worth noting that the electrocatalytic HER performance over the resultant Mo-Cu_2_S was better than that of most of previously reported Cu_2_S-based electrocatalyst for HER (Table S2) [26,27,41,42,43,44,45,46,47]. Furthermore, the HER kinetics of electrocatalysts are studied in the reference of Tafel slopes in Figure 5B. The Tafel slope of Mo-Cu_2_S/CF is 171 mV dec^−1^, smaller than that of Cu_2_S/CF (181 mV dec^−1^) and pure CF (420 mV dec^−1^), suggesting that Mo-Cu_2_S/CF proceeds the rapidest HER kinetics among them. Electrochemical impedance spectroscopy (EIS) is then examined to reveal electron transfer rates during the HER process. The corresponding Nyquist plots are shown in Figure 5C. Mo-Cu_2_S/CF exhibits a smaller charge transfer resistance compared to Cu_2_S/CF and pure CF, which indicates that Mo-Cu_2_S/CF shows the faster electron transfer and HER kinetics than Cu_2_S/CF and pure CF. Electrochemical specific surface area (ECSA) is also an important parameter to estimate the electrochemical properties, which is proportional to double-layer capacitance (C_dl_). The C_dl_ values of different electrodes are calculated by fitting of the plots of current density at different scan rates. As manifested in Figure 5D, the C_dl_ of Mo-Cu_2_S/CF (58 mF cm^−2^) is much higher than that of Cu_2_S/CF (50 mF cm^−2^) and pure CF (0.04 mF cm^−2^), meaning that Mo-Cu_2_S/CF possess more active surface area, generally resulting in even more active sites and normally better catalytic activity [48].

Meanwhile, the highly durability of Mo-Cu_2_S/CF is investigated by chronopotentiometry, and Mo-Cu_2_S/CF displays a negligible attenuation of overpotential after 20 h at 60 mA cm^−2^, proving its excellent stability. The XRD pattern in Figure S5 signifies that the phase remains Mo-Cu_2_S after 20 h HER electrocatalysis. Meanwhile, the TEM, HRTEM and elementals mapping images after 20 h HER test (Figure S6), the microstructure and composition of the catalyst were well-maintained, confirming the outstanding structural stability after long-term HER electrocatalysis.

From the above tests, it is inferred that the excellent HER activity of Mo-Cu_2_S/CF has the following three aspects: (I) using foam copper (CF) as the substrate is conducive to the uniform distribution of Mo-Cu_2_S on the surface, and can effectively inhibit the interlayer aggregation of the nanosheets. Besides, the “all in one” self-supporting electrode improves the catalytic stability and activity avoids the influence of the binder between the catalyst surface and the electrolyte. (II) multilayer nanosheets of Mo-Cu_2_S/CF increases the contact area with electrolyte in the process of HER; this structure provides more active sites, which makes the hydrogen evolution reaction more efficient. (III) Mo doping increases the charge transfer rate, which is beneficial to the electrocatalytic process.

## 3. Experimental Section

### 3.1. Reagents and Materials

Sodium molybdate dishydrate (NaMoO_4_·2H_2_O) and thioacetamide (C_2_H_5_NS) were analytical grade and bought from Sinopharm Group Chemical Reagent Co., Ltd. (Beijing, China). KOH, C_3_H_2_O and CH_3_CH_2_OH were purchased from Tianjin Kemeiou Reagent Co., Ltd. (Tianjin, China) The copper foam (CF, with 1 mm thickness) was obtained from Suzhou Jiashide foam metal Co., Ltd. (Suzhou, China).

### 3.2. Synthesis of Mo-Cu_2_S/CF Samples

The Mo-Cu_2_S/CF samples were prepared by one-step hydrothermal method. Typically, 0.25 mmol of Na_2_MoO_4_·2H_2_O and 1.25 mmol of C_2_H_5_NS were dissolved in 30 mL ultrapure water under stirring treatment. A piece of 1 cm × 5.5 cm copper foam (CF) was cleaned with acetone and 3 M hydrochloric acid for 15 min in each to remove the impurities on the surface. It was then immersed in ethanol and deionized water 5 min respectively to wash several times alternately. Subsequently, the obtained mixture was added into a 50 mL polyphenylene autoclave, which was heated at 180 °C oven for 24 h, and then cooled to indoor temperature. Finally, the resulting materials was obtained and repeatedly rinsed with water and ethanol several times, subsequently dried at 60 °C for 10 h in vacuum. In addition, so as to further investigate the impact of hydrothermal reaction time on the structure and catalytic performance, three other samples by changing the reaction time (6 h, 12 h and 36 h) were synthesized, which were denoted as Mo-Cu_2_S/CF -6, Mo-Cu_2_S/CF -12 and Mo-Cu_2_S/CF -36.

### 3.3. Synthesis of Cu_2_S/CF Samples

In the synthesis of Cu_2_S/CF, the reaction conditions were the same as those of Mo-Cu_2_S/CF, except that Na_2_MoO_4_·2H_2_O was not added in the raw material.

### 3.4. Electrochemical Measurements

Relevant electrochemical measurements were conducted in a typical three-electrode electrochemical system and performed on the CHI660E B17060 electrochemical workstation (Chenhua Instrument Co., LTD. Shanghai). The as-prepared Mo-Cu_2_S/CF and Cu_2_S/CF served as the working electrode, as well as extremely saturated calomel reference electrode (SCE) and graphite carbon counter electrode. For the electrochemical measurements, the prepared Mo-Cu_2_S/CF material exposed a 0.3 cm × 0.4 cm area as a working electrode. The HER measurements were carried out in 1 M KOH, and the potential vs. SCE was converted into reversible hydrogen electrode (RHE) according with the equation of E*_vs_* _RHE_ = E*_vs_* _SCE_ + 0.242 + 0.059 pH. The linear sweep voltammetry (LSV) curve was performed at the scan rate of 5 mV s^−1^ and iR-corrected. Tafel slopes were derived from the LSV curves. Electrochemical impedance spectroscopy (EIS) was measured at a voltage generated by Faraday current with a frequency range from 10^−2^ to 10^5^ Hz. The stability measurement was evaluated with I-T curve under a constant voltage. Electrochemical specific surface area (ECSA) was obtained by conducting cyclic voltammetry (CV) under the different scanning speeds (20, 40, 60, 80, 100 and 120 mV s^−1^), which is proportional to the C*_dl_* and also an important factor in electrochemical measurements.

### 3.5. Materials Characterization

X-ray diffraction (XRD) data were performed by the Rigaku D/max-2200pc. The microstructure and morphology were monitored by field emission scanning electron microscopy (FESEM, Hitachi, S4800), the transmission electron microscopy and high-resolution TEM were texted on Tecnai G2 F20S-TWIN. X-ray photoelectron spectroscopy (XPS) was obtained on an XIS SUPRA.

## 4. Conclusions

In summary, a novel Mo-doped Cu_2_S nanosheets (Mo-Cu_2_S/CF) grown in situ on copper foam (CF) has been successfully synthesized by a simple one-step hydrothermal method. Experimental results provide evidence that Mo doping can regulate the catalyst morphology and modulate the electronic structure, thus enhancing the hydrogen evolution activity. The over potential is only 322 mV in the alkaline condition (1 m KOH) at current density 100 mA cm^−2^, and the stability could be maintained for at least 20 h. Therefore, the optimized Mo-Cu_2_S/CF catalyst exhibiting remarkable electrocatalytic activity provides a direction for the development of transition metal sulfide self-supported electrode for HER.

## Supplementary Materials

The following supporting information can be downloaded at: https://www.mdpi.com/article/10.3390/molecules27185961/s1, Figure S1. XRD of Mo-Cu_2_S/CF-6 h; Mo-Cu_2_S/CF-12 h; Mo-Cu_2_S/CF-24 h; Mo-Cu_2_S/CF-36 h, Figure S2. (A–C) SEM, TEM and HRTEM images, (D–F) The corresponding elementals mapping images of Cu_2_S/CF, Figure S3. STEM-EDX spectrum of the Mo-Cu_2_S/NF, Figure S4. Polarization curves of Mo-Cu_2_S/CF-6 h; Mo-Cu_2_S/CF-12 h; Mo-Cu_2_S/CF-24 h; Mo-Cu_2_S/CF-36 h, Figure S5. XRD of Mo-Cu_2_S/CF after HER test, Figure S6. (A–B) TEM and HRTEM images, (C–F) The corresponding elementals mapping images of Mo-Cu_2_S/CF after 20 h HER test, Table S1. The atomic percentage of the Mo-Cu_2_S/NF, Table S2. Comparison of the electrocatalytic activity of Mo-Cu_2_S with previously reported Cu_2_S-based electrocatalysts in 1.0 M KOH electrolyte.
