# Supplementary material for: Mo-Doped Cu2S Multilayer Nanosheets Grown In Situ on Copper Foam for Efficient Hydrogen Evolution Reaction
Source: Molecules. 2022 Sep 13;27(18):5961. doi: 10.3390/molecules27185961 (PMC9501039; doi:10.3390/molecules27185961)
Supplement: Supplementary file 1 [file molecules-27-05961-s001.zip › molecules-1905272-supplementary.pdf]

Supplementary Material

Mo Doped Cu_2S Multilayer Nanosheets In Situ Grown on the Copper Foam for Efficient Hydrogen Evolution Reaction

Yajie Xie [1,2], Jianfeng Huang [1,2,*], Rui Xu [2], Danyang He [2], Mengfan Niu [2], Xiaoyi Li [2], Guoting Xu [2], Liyun Cao [2] and Liangliang Feng [1,2,*]

[1] Key Laboratory of Auxiliary Chemistry and Technology for Chemical Industry, Ministry of Education, Shaanxi University of Science and Technology, Xi'an 710021, China
[2] School of Material Science and Engineering, International S&T Cooperation Foundation of Shaanxi Province, Shaanxi University of Science and Technology, Xi'an 710021, China
* Correspondence: huangjf@sust.edu.cn (J.H.); fengll@sust.edu.cn (L.F.)

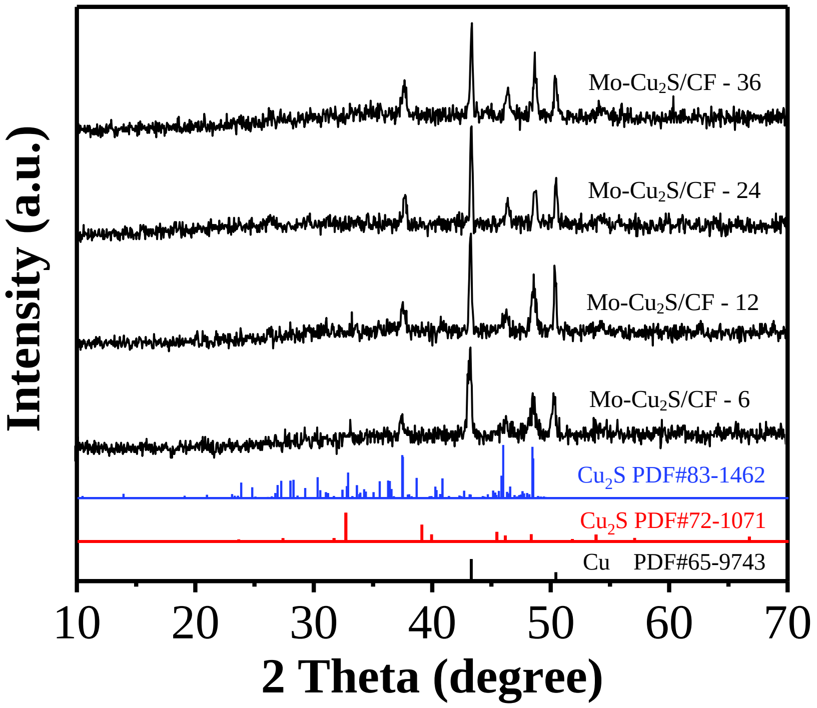

Figure S1. XRD of Mo-Cu_2S/CF-6h; Mo-Cu_2S/CF-12h; Mo-Cu_2S/CF-24h; Mo-Cu_2S/CF-36h.

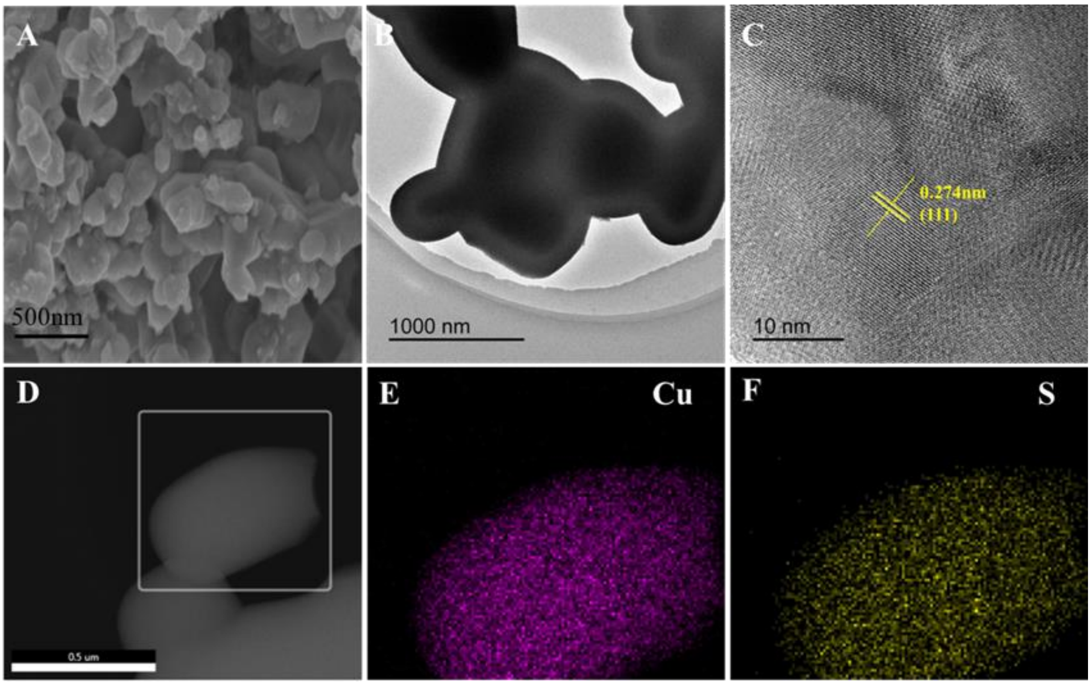

Figure S2. (**A–C**) SEM, TEM and HRTEM images, (**D–F**) the corresponding elementals mapping images of Cu_2S/CF.

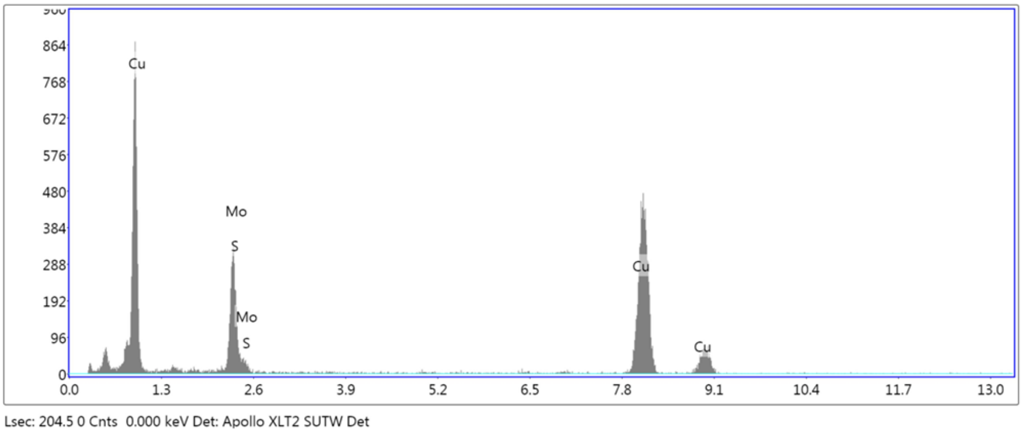

Figure S3. STEM-EDX spectrum of the Mo-Cu_2S/NF.

Table S1. The atomic percentage of the Mo-Cu_2S/NF.

Element	Weight %	Atomic %	Net Int.	Error %	Kratio	Z	R	A	F
S K	58.78	74.23	8.5	10.04	0.21	1.03	0.96	0.35	1.00
CuK	38.92	24.80	36.2	5.56	0.22	0.95	1.06	0.58	1.00
MoK	2.30	0.97	1.1	21.92	0.02	0.94	1.16	0.75	1.00

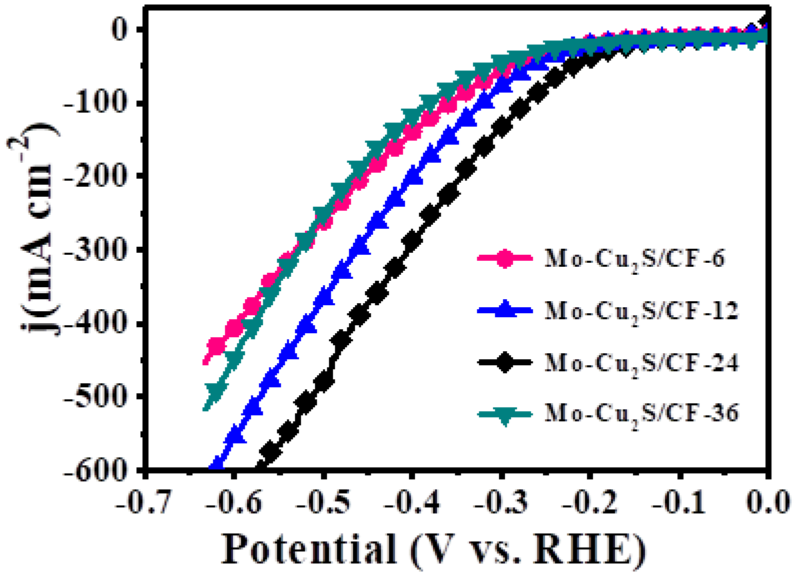

Figure S4. Polarization curves of Mo-Cu_2S/CF-6h; Mo-Cu_2S/CF-12h; Mo-Cu_2S/CF-24h; Mo-Cu_2S/CF-36h.

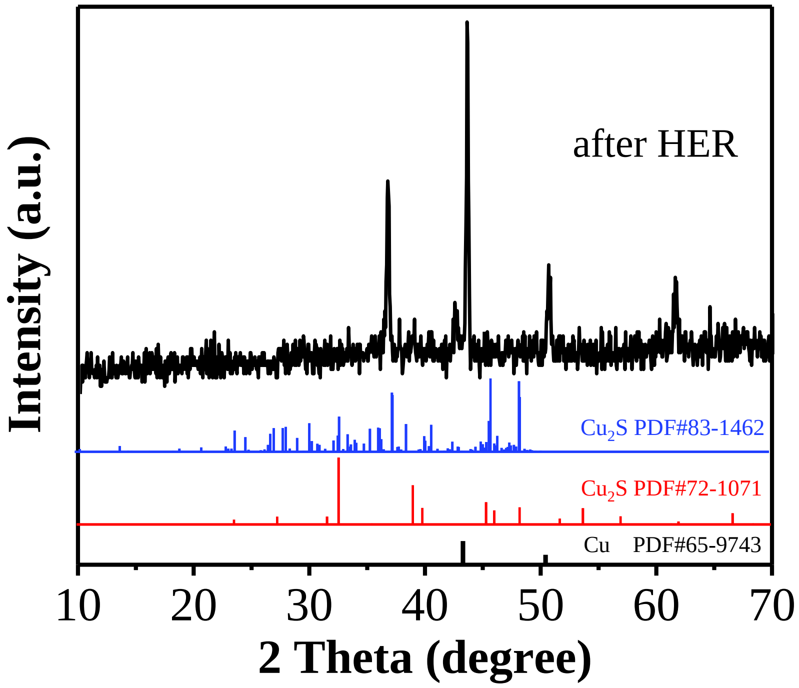

Figure S5. XRD of Mo-Cu_2S/CF after HER test.

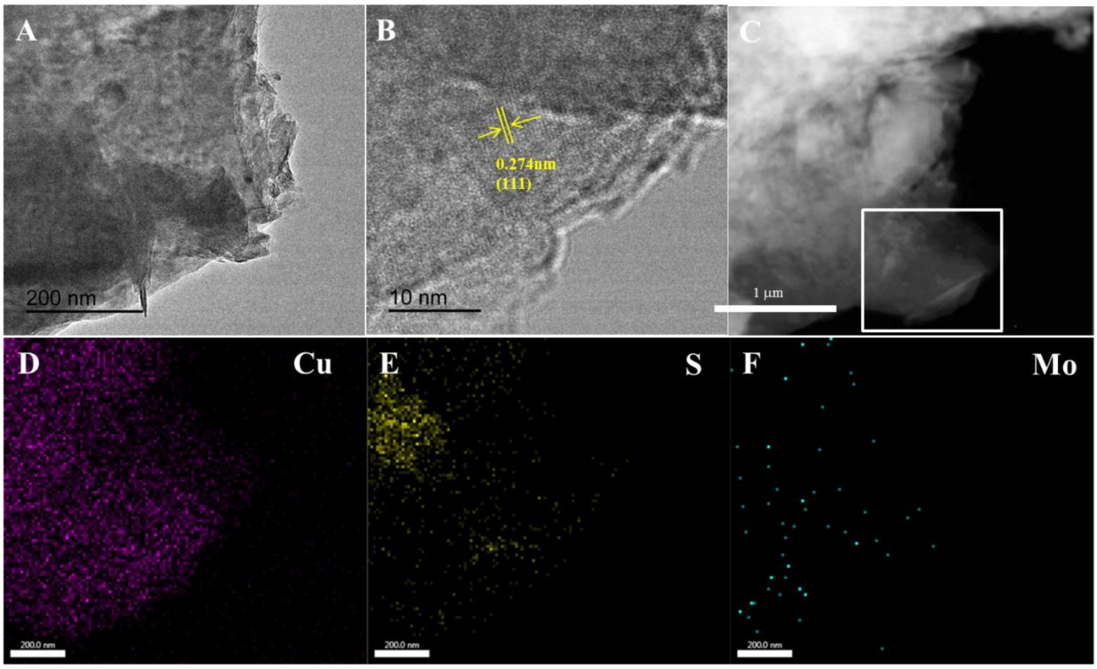

Figure S6. (**A–B**) TEM and HRTEM images, (**C**) the STEM image, (**D–F**) the corresponding elementals mapping images of Mo-Cu_2S/CF after 20 h HER test.

Table S2. Comparison of the electrocatalytic activity of Mo-Cu_2S with previously reported Cu_2S-based electrocatalysts in 1.0 M KOH electrolyte.

**Catalysts**	**Electrolyte**	**Current Density (j, mA/cm^2)**	**Overpotential at Corresponding j (mV)**	**Reference**
Mo-Cu_2S/CF	1.0 M KOH	10	18	This work
	1.0 M KOH	100	322	
Cu_2S films	1.0 M KOH	10	445	Fuel 2022,322,124073 [41]
V-Cu_2S-Nanowires NW	1.0 M KOH	10	188	Scr. Mater. 2021, 196, 113756 [27]
Cu_2S HNAs-CF	1.0 M KOH	100	125	Sustain. Energy Fuels 2021, 5, 2633–2639 [42]
Cu_2S@Ni_3Se_2	1.0 M KOH	10	106	J. Colloid Interface Sci. 2021, 592, 13–21 [43]
Cu_2S-Ni_3S_2/NF	1.0 M KOH	10	149	Chemistry Select 2020, 5, 2455–2464 [44]
Cu_2S@Cu	1.0 M KOH	10	316	Int. J. Hydrogen Energy 2019, 44, 1620–1626 [26]
Cu_2S-$Co(OH)_2$ NTA/CF	1.0 M KOH	50	241	Electrochim. Acta 2019, 316, 8–18 [45]
Cu2S NRs@CoS/CF)	1.0 M KOH	50	235	Electrochim. Acta 2018, 296, 1035–1041 [46]
Co-Cu_2S/CF	1.0 M KOH	20	298	J. Alloys Compd. 2018, 762, 637–642 [47]
	1.0 M KOH	50	357	
